# Association between air pollutants and initiation of biological therapy in patients with ankylosing spondylitis: a nationwide, population-based, nested case–control study

**DOI:** 10.1186/s13075-023-03060-4

**Published:** 2023-05-05

**Authors:** Chung-Mao Kao, Yi-Ming Chen, Wen-Nan Huang, Yi-Hsing Chen, Hsin-Hua Chen

**Affiliations:** 1 Division of Allergy, Immunology, and Rheumatology, Department of Internal Medicine, Taichung Veterans General Hospital, No.1650, Sec.4, Taiwan Boulevard, Taichung, 40705 Taiwan; 2 Division of Translational Medicine, Department of Medical Research, Taichung Veterans General Hospital, Taichung, Taiwan; 3 School of Medicine, National Yang Ming Chiao Tung University, Taipei, Taiwan; 4 Department of Post-Baccalaureate Medicine, College of Medicine, National Chung Hsing University, Taichung, Taiwan; 5 Institute of Biomedical Science and Rong Hsing Research Center for Translational Medicine, National Chung Hsing University, Taichung, Taiwan; 6 Department of Business Administration, Ling-Tung University, Taichung, Taiwan; 7 Department of Industrial Engineering and Enterprise Information, Tunghai University, Taichung, Taiwan; 8 Institute of Public Health and Community Medicine Research Center, National Yang Ming Chiao Tung University, Taipei, Taiwan; 9 Big Data Center, National Chung Hsing University, Taichung, Taiwan; 10 Division of General Medicine, Department of Internal Medicine, Taichung Veterans General Hospital, Taichung, Taiwan

**Keywords:** Air pollutants, Biologics, Ankylosing spondylitis, Nested case–control study

## Abstract

**Background:**

Outdoor air pollution has been found to trigger systemic inflammatory responses and aggravate the activity of certain rheumatic diseases. However, few studies have explored the influence of air pollution on the activity of ankylosing spondylitis (AS). As patients with active AS in Taiwan can be reimbursed through the National Health Insurance programme for biological therapy, we investigated the association between air pollutants and the initiation of reimbursed biologics for active AS.

**Methods:**

Since 2011, hourly concentrations of ambient air pollutants, including PM2.5, PM10, NO2, CO, SO2, and O3, have been estimated in Taiwan. Using Taiwanese National Health Insurance Research Database, we identified patients with newly diagnosed AS from 2003 to 2013. We selected 584 patients initiating biologics from 2012 to 2013 and 2336 gender-, age at biologic initiation-, year of AS diagnosis- and disease duration-matched controls. We examined the associations of biologics initiation with air pollutants exposure within 1 year prior to biologic use whilst adjusting for potential confounders, including disease duration, urbanisation level, monthly income, Charlson comorbidity index (CCI), uveitis, psoriasis and the use of medications for AS. Results are shown as adjusted odds ratio (aOR) with 95% confidence intervals (CIs).

**Results:**

The initiation of biologics was associated with exposure to CO (per 1 ppm) (aOR, 8.57; 95% CI, 2.02–36.32) and NO2 (per 10 ppb) (aOR, 0.23; 95% CI, 0.11–0.50). Other independent predictors included disease duration (incremental year, aOR, 8.95), CCI (aOR, 1.31), psoriasis (aOR, 25.19), use of non-steroidal anti-inflammatory drugs (aOR, 23.66), methotrexate use (aOR, 4.50; 95% CI, 2.93–7.00), sulfasalazine use (aOR, 12.16; 95% CI, 8.98–15.45) and prednisolone equivalent dosages (mg/day, aOR, 1.12).

**Conclusions:**

This nationwide, population-based study revealed the initiation of reimbursed biologics was positively associated with CO levels, but negatively associated with NO_2_ levels. Major limitations included lack of information on individual smoking status and multicollinearity amongst air pollutants.

**Supplementary Information:**

The online version contains supplementary material available at 10.1186/s13075-023-03060-4.

## Introduction

Ankylosing spondylitis (AS) is a systemic disease characterised by chronic inflammation of axial and peripheral joint and subsequent ankylosis, resulting in an impairment of productivity and activity of living, as well as an increasing socioeconomic burden [1, 2]. AS in Taiwan features a male predominance, with the standardised prevalence being 0.24% in 2010 and an incidence rate of 0.42–0.50 cases per 1000 person-years [3]. Early intervention is essential in controlling inflammation through first-line pharmacological treatment, using non-steroidal anti-inflammatory drugs (NSAID) for spinal and peripheral involvement and conventional synthetic disease-modifying antirheumatic drugs for peripheral arthritis. For AS patients showing an inadequate response to first-line therapy, the use of biologics, including tumour necrosis factor inhibitors and interleukin-17 inhibitors, is indicated [4]. AS patients in Taiwan can access biologics through reimbursement of NHI programme or through self-funded use. The therapy can be used through NHI reimbursement if they have two records of high disease activity at least 4 weeks apart, defined as the simultaneous presence of serum levels of erythrocyte sedimentation rate > 28 mm/h, C-reactive protein > 1.0 mg/dl, and Bath Ankylosing Spondylitis Disease Activity Index ≥ 6 though having undergone the following treatments: (i) full doses of at least two different NSAID therapies over 4 weeks respectively at the same medical institution for at least 3 months unless intolerance; or (ii) concomitant use of NSAIDs and sulfasalazine at a dose of 2 g per day over 4 months unless intolerance for peripheral arthritis.

Outdoor air pollution has been found to trigger systemic inflammatory responses [5], and long-term exposure to air pollution is associated with an increased risk of development or aggravation of certain immune-mediated diseases [6, 7]. Long-term exposure to particulate matter < 2.5 μm in size (PM2.5) has been found strongly correlated with worse AS outcomes [8]. However, whether air pollutants other than PMs, including carbon monoxide (CO), sulphur dioxide (SO_2_), ozone (O_3_) and nitrogen dioxide (NO_2_), are associated with AS disease activity remains uncertain. As the harm of air pollution to rheumatic diseases is becoming an issue of concern for rheumatologists globally, a preliminary large-scale census is imperative. In Taiwan, the National Health Insurance Research Database (NHIRD) has facilitated longitudinal epidemiologic studies. Also, a database of estimated levels of ambient air pollutants is available and can be linked to the NHIRD. Therefore, we investigated the association between levels of air pollutants with the initiation of biologics, a proxy for high activity, in patients with AS using the NHIRD linking to database of ambient air pollutant levels.

## Materials and methods

### Study design

This was a nationwide, population-based, nested case–control study.

### Source of data

The claims data on AS study subjects were obtained from the NHIRD from January 1, 2003, to December 31, 2013. An obligatory, single-payer NHI programme covering more than 99% of Taiwan’s population was implemented on March 1, 1995 [9], and has facilitated population-based longitudinal epidemiologic studies. The NHIRD includes claims data concerning demographics, outpatient and admission services, medical expenditures covered by NHI, diagnoses and procedures with corresponding International Classification of Diseases-Ninth Revision-Clinical Modification (ICD-9-CM) codes, and medication prescriptions with corresponding Anatomical Therapeutic Chemical Classification codes, whilst lacking certain personal data, including weight, alcohol and tobacco use, and AS disease activity, damage and functional measures.

### Estimated mean levels of the exposed ambient air pollutants

Since 2011, the concentrations of various ambient air pollutants at 374 residential locations in Taiwan, except for Kinmen and Mazu, have been estimated using a spatio-temporal model built by a deep-learning approach [10]. This model has assessed the concentrations of air pollutants at each location using a graph convolutional neural network, with the levels at each location calculated based on data taken from three air quality censoring stations near the specified location. We used the hourly concentrations of air pollutants across 60 air quality censoring stations to estimate the mean levels of air pollutants, including PM2.5, PM10, NO_2_, CO, SO2 and O_3_, within 1 year before index dates [11].

### Study subjects

In Taiwan, AS was diagnosed based on either 1984 modified New York criteria [12] or 2009 Assessment of SpondyloArthritis International Society classification criteria for axial spondyloarthritis [13, 14]. In this study, newly diagnosed AS patients aged ≥ 20 years were identified as those having at least three ambulatory visits with an AS diagnosis (ICD-9-CM code 720.0) and a concurrent prescription of NSAIDs, corticosteroids, sulfasalazine or methotrexate from 2003 to 2013. Patients who also had outpatient or inpatient visits with a diagnosis of rheumatoid arthritis (ICD-9-CM code 714.0) and who used biologics, including etanercept, adalimumab and golimumab before the first date of outpatient or inpatient visit with AS diagnosis were excluded. Patients with history of biologics use not within the NHI reimbursement period for active AS (i.e. use of adalimumab before August 1, 2009, etanercept before November 1, 2009, and golimumab before January 1, 2012) or biologics without NHI reimbursement (i.e. use of biologics other than adalimumab, etanercept and golimumab), and those without outpatient visits after 2009 were excluded, in order to ensure all biologics users were prescribed the approved biologics for active AS reimbursed by NHI. Those residing in Kinmen and Mazu were excluded to ensure the estimated air pollutant levels at all subjects’ residential locations could be obtained.

### Selection of biologics case group and matched control group

From the abovementioned population, subjects who initiated NHI-reimbursed biologics were identified as the biologics case group, and those who did not as the control group. All subjects were designated specific index dates, which was the date of first NHI-reimbursed biologics initiation for biologics cases and date of first outpatient department visit each year for controls. After matching for the year of index dates between the two groups, we then examine whether exposure to air pollutants within 1 year before first NHI-reimbursed biologics initiation for active AS influenced patients’ disease activity. We matched subjects in both groups at a 1:4 ratio for gender, age at first NHI-reimbursed biologic initiation (± 3 years), year of first AS diagnosis, and AS disease duration (± 0.3 years) to adjust for confounding effects [15].

**Fig. 1:**
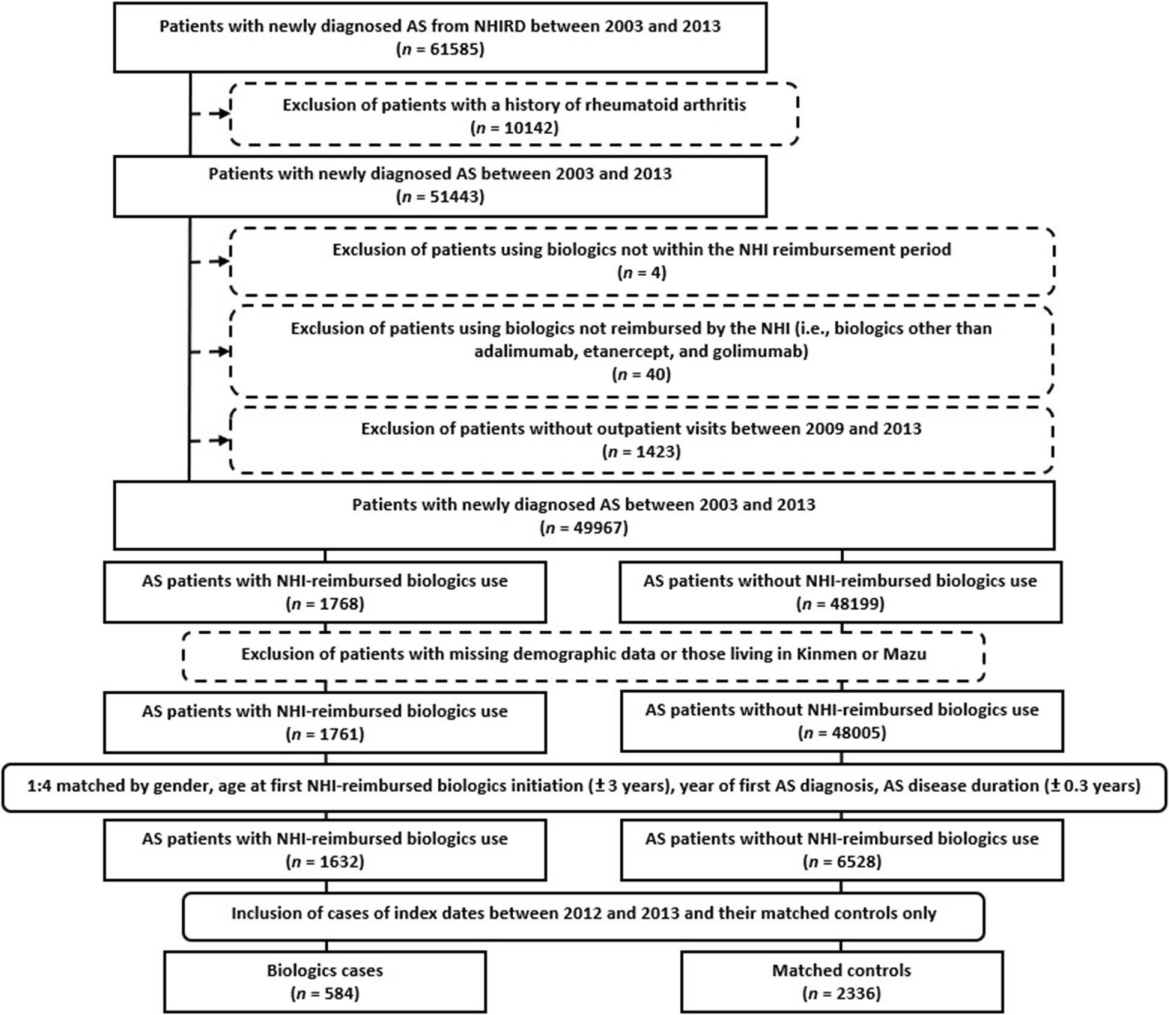
Flow diagram of enrollment, categorisation and matching for comparison of study subjects. For AS patients in Taiwan, the start of biologic use under NHI reimbursement was August 1, 2009, for adalimumab; November 1, 2009, for etanercept; and January 1, 2012, for golimumab. Amongst NHI-reimbursed biologics users, 1036 patients initiated with adalimumab, 614 with etanercept, and 118 with golimumab. The index date is the date of the first NHI-reimbursed biologics initiation for biologics cases and the date of first outpatient department visits each year for controls. Age at first NHI-reimbursed biologics initiation, or age at index date, is regarded as the patient’s age in this study. AS disease duration indicates the period between first diagnosis of AS and first NHI-reimbursed biologics initiation (index date). AS, ankylosing spondylitis; NHI, National Health Insurance; NHIRD, National Health Insurance Research Database

Due to the available estimated levels of exposed air pollutants gathered from 2011, we included subjects with index dates between 2012 and 2013 only for final analyses. The process for the selection of study subjects for final analysis is depicted in Fig. 1.

### Potential confounders

Potential confounders existing within 1 year before index date to be adjusted included socioeconomic status, represented by monthly income and urbanisation level of residence, comorbidities, extra-articular manifestations, and use of medications for AS. Four clusters of urbanisation levels were categorised according to the density of population (people/km^2^), population ratio of those aged ≥ 65 years, population ratio of agricultural workers and number of physicians per 100,000 individuals, with level 1 indicating the most urbanised districts and level 4 the least [16]. Four clusters of monthly income were stratified based on quartiles. Charlson Comorbidity Index (CCI) at the year of index date was used to represent patients’ general comorbid condition [17]. The presence of comorbidities used to calculate the CCI was defined as the occurrence of ≥ 3 outpatient visits or ≥ 1 inpatient visit with a corresponding ICD-9-CM code within one year before index date. Extra-articular manifestations, including uveitis, psoriasis and inflammatory bowel disease, were diagnosed by corresponding specialists and recognised by their ICD-9 codes, with those diagnosed more than 1 year before index date being excluded. The medications involved included NSAIDs, methotrexate, sulfasalazine and systemic corticosteroids and were identified by their Anatomical Therapeutic Chemical classification codes. The impact of daily dosage of corticosteroids, shown as prednisolone equivalent dose, on biologics initiation was also adjusted. The summary of included diseases, manifestations, medications and their corresponding codes is given in Supplementary Table 1, Additional file 1.

After adjusting for the above potential confounders through conditional logistic regression analyses, we examined the association between levels of air pollutants and biologics initiation for active AS.

### Sensitivity analysis

We also conducted analyses using the time horizon of within 3 months before index date for the sensitivity analysis.

### Statistical analyses

Continuous variables are shown as mean ± standard deviation and categorical variables as number (percentage) of patients. Between groups, we compared continuous variables by the independent *t*-test and categorical variables by chi-square test or Fisher’s exact test.

Because the impact of air pollutants might have multicollinearity and correlation based on their chemical properties, a correlation table including variance inflation factors (VIF) of each air pollutant amongst all pollutants and Pearson correlation coefficients with *p*-values of all pairs of air pollutants was plotted. VIF ≥ 10 was considered significant multicollinearity necessitating being corrected.

We examined the association between biologics initiation and levels of exposed air pollutants using multivariable conditional logistic regression models for adjustment, shown as adjusted odds ratios (aORs) with 95% confidence intervals (CIs). A probability (*p*) of < 0.05 was considered statistically significant. Statistical calculations were performed using the Statistical Package for the Social Sciences (SPSS), Windows Version 13.0 (SPSS Inc., Chicago, IL, USA).

## Results

### Baseline characteristics of the study population

The flow diagram regarding enrollment, categorisation and matching for comparisons in the study population is shown in Fig. 1. After the process surrounding the selection of study subjects for final analysis, we enrolled 2920 AS patients, consisting of 584 patients who initiated NHI-reimbursed biologics as the cases, including 237 with etanercept, 242 with adalimumab and 105 with golimumab, along with 2336 patients who did not as the controls (Table 1 and Supplementary Table 2, Additional file 1). The study population was predominantly composed of male subjects (*n* = 2,455, 84.1%). The mean age at first NHI-reimbursed biologic initiation was 40.2 ± 12.8 years in the cases and 40.4 ± 12.5 in the controls (*p* = 0.77). The disease duration of AS was 6.1 ± 3.5 years for the cases and 6.0 ± 3.5 for the controls (*p* = 0.44).

**Table 1:** Baseline characteristics amongst matched study subjects with and without initiation of reimbursed biologics

	**Controls** **(*n* = 2336)**	**Biologics cases** **(*n* = 584)**	***p*-value**
**Gender**			1.00
Female, *n* (%)	372 (15.9)	93 (15.9)	
Male, *n* (%)	1964 (84.1)	491 (84.1)	
**Age at first NHI-reimbursed biologic initiation (years)**, mean ± S.D	40.4 ± 12.5	40.2 ± 12.8	0.77
**Disease duration (years)**, mean ± S.D	6.0 ± 3.5	6.1 ± 3.5	0.44
**Monthly income (NTD)**			0.70
≤ 15,840, *n* (%)	656 (28.1)	176 (30.1)	
15,841–28,800, *n* (%)	812 (34.8)	204 (34.9)	
28,801–45,800, *n* (%)	478 (20.5)	115 (19.7)	
≥ 45,801, *n* (%)	390 (16.7)	89 (15.2)	
**Urbanisation**			0.01
Level 1, *n* (%)	757 (32.4)	158 (27.1)	
Level 2, *n* (%)	738 (31.6)	181 (31.0)	
Level 3, *n* (%)	416 (17.8)	109 (18.7)	
Level 4, *n* (%)	425 (18.2)	136 (23.3)	
**CCI at the year of index date,** mean ± S.D	0.2 ± 0.7	0.4 ± 0.8	< 0.01
CCI = 0, *n* (%)	1994 (85.4)	405 (69.3)	
CCI ≥ 1, *n* (%)	342 (14.6)	179 (30.7)	< 0.01
**Extra-articular manifestations**			
Uveitis, *n* (%)	59 (2.5)	42 (7.2)	< 0.01
Psoriasis, *n* (%)	10 (0.4)	42 (7.2)	< 0.01
Inflammatory bowel disease, *n* (%)	7 (0.3)	7 (1.2)	0.01
**Medications**			
NSAIDs, *n* (%)	1663 (71.2)	579 (99.1)	< 0.01
Methotrexate, *n* (%)	50 (2.1)	124 (21.2)	< 0.01
Sulfasalazine, *n* (%)	656 (28.1)	506 (86.6)	< 0.01
Corticosteroids, *n* (%)	492 (21.1)	292 (50.0)	< 0.01
Corticosteroids, prednisolone equivalent dose (mg/day), mean ± S.D	0.2 ± 1.9	1.2 ± 2.8	< 0.01
**Exposed levels of ambient air pollutants**			
PM2.5 (per 10 μg/m^3^), mean ± S.D	3.0 ± 0.6	3.0 ± 0.6	0.38
PM10 (per 10 μg/m^3^), mean ± S.D	5.2 ± 1.2	5.2 ± 1.1	0.78
SO_2_ (per 10 ppb), mean ± S.D	0.4 ± 0.1	0.3 ± 0.1	0.01
NO_2_ (per 10 ppb), mean ± S.D	1.9 ± 0.6	1.8 ± 0.5	< 0.01
CO (per 1 ppm), mean ± S.D	0.6 ± 0.2	0.5 ± 0.2	0.02
O_3_ (per 10 ppb), mean ± S.D	2.8 ± 0.3	2.8 ± 0.3	0.08

A *p*-value < 0.05 is considered statistically significant. *CCI* Charlson comorbidity index, *NHI* National Health Insurance, *NSAIDs* Nonsteroidal anti-inflammatory drugs, *NTD* New Taiwan dollar, *PM* Particulate matter, *S.D.* Standard deviation

Regarding socioeconomic status, the monthly incomes subjects received were independent of requirements for biologics therapy (*p* = 0.70). With regard to levels of urbanisation, a lower proportion of biologics cases resided in more urbanised districts (*p* = 0.01).

The CCIs at the year of index date were 0.4 ± 0.8 in the cases and 0.2 ± 0.7 in the controls (*p* < 0.01), with a higher proportion of subjects with CCIs ≥ 1 seen in the cases (*p* < 0.01), indicating that AS patients initiating biologics therapy suffered from more comorbidities. Higher proportions of subjects in the cases presented with extra-articular manifestations, including uveitis (*p* < 0.01), psoriasis (*p* < 0.01), and inflammatory bowel disease (*p* = 0.01) within 1 year before their biologics initiation.

Biologics cases possessed higher proportions of subjects taking NSAIDs (*p* < 0.01), methotrexate (*p* < 0.01), sulfasalazine (*p* < 0.01) and systemic corticosteroids *p* < 0.01), with higher prednisolone equivalent doses as well (*p* < 0.01) before biologics initiation.

Subjects in the cases were exposed to lower levels of ambient SO_2_ (*p* = 0.01), NO_2_ (*p* < 0.01) and CO (*p* = 0.02), than those in the controls.

### Effect of multicollinearity for air pollutant levels

The correlation table showing the levels of multicollinearity for all exposed air pollutant levels and levels of correlation for all pairs of them within 1 year before index date was presented in Table 2. Significant effect of multicollinearity was found for NO_2_ (VIF = 11). With respect to NO_2_, a significantly positive correlation was found between CO and NO_2_ levels (correlation coefficient: 0.904). The other pair bearing high correlation was PM2.5 and PM10, but the VIFs of both were not demonstrated with significant multicollinearity.

**Table 2:** Correlation table for ambient air pollutant levels within one year before index date

**(*n* = 2920)**	**VIF**	PM2.5 (per 10 μg/m^3^)	PM10 (per 10 μg/m^3^)	SO_2_ (per 10 ppb)	NO_2_ (per 10 ppb)	CO (per 1 ppm)	O_3_ (per 10 ppb)
PM2.5 (per 10 μg/m^3^)	6	1	0.908	0.487	0.052	− 0.024	0.188
PM10 (per 10 μg/m^3^)	7	< 0.01	1	0.518	− 0.054	− 0.091	0.301
SO_2_ (per 10 ppb)	2	< 0.01	< 0.01	1	0.253	0.076	0.007
NO_2_ (per 10 ppb)	11	< 0.01	< 0.01	< 0.01	1	0.904	− 0.760
CO (per 1 ppm)	7	0.20	< 0.01	< 0.01	< 0.01	1	− 0.670
O_3_ (per 10 ppb)	3	< 0.01	< 0.01	0.71	< 0.01	< 0.01	1

The VIF of each air pollutant indicates the level of multicollinearity with others, and VIF ≥ 10 is considered significant multicollinearity necessitating being corrected. For each pair of air pollutants, Pearson’s correlation coefficient is presented from the right upper part, and the *p*-value from the left lower part of the table. A *p*-value < 0.05 is considered statistically significant. *PM* Particulate matter, *VIF* Variance inflation factor

A correlation table using the time horizon of within 3 months before index date was demonstrated in Supplementary Table 3, Additional file 1, showing consistent results with those in Table 2.

### Association between exposed levels of air pollutants and high AS activity necessitating initiation of NHI-reimbursed biologics

The results from multivariable conditional logistic regression analyses are presented as model 1 in Table 3. Initiation of biologics for active AS was not associated with age at biologics initiation, socioeconomic status or diagnosis of uveitis and inflammatory bowel disease within 1 year before biologics initiation. It was however positively associated with disease duration, CCI at year of index date, diagnosis of psoriasis and use of NSAIDs, methotrexate, sulfasalazine and systemic corticosteroids in a dose-dependent manner within 1 year before biologics initiation.

**Table 3:** Association between initiation of reimbursed biologics and air pollutants in adjustment for potential confounders

	**Univariable analysis**		**Multivariable analysis** **(model 1)**		**Multivariable analysis** **(model 2**^a^**)**		**Multivariable analysis** **(model 3**^b^**)**	
	**Odds ratio** **(95% CI)**	***p*-value**	**Adjusted odds ratio** **(95% CI)**	***p*-value**	**Adjusted odds ratio** **(95% CI)**	***p*-value**	**Adjusted odds ratio** **(95% CI)**	***p*-value**
**Age at first NHI-reimbursed biologic initiation**	1.00 (0.99–1.01)	0.76	1.00 (0.99–1.01)	0.74	1.00 (0.99–1.01)	0.77	1.00 (0.99–1.01)	0.82
**Disease duration**	3.22 (2.42–4.27)	< 0.01	8.95 (5.97–13.42)	< 0.01	9.25 (6.18–13.86)	< 0.01	9.19 (6.14–13.77)	< 0.01
**Monthly income (NTD)**								
≤ 15,840	1 (Reference)		1 (Reference)		1 (Reference)		1 (Reference)	
15,841–28,800	0.94 (0.75–1.17)	0.56	0.79 (0.59–1.07)	0.13	0.81 (0.60–1.09)	0.16	0.80 (0.59–1.08)	0.14
28,801–45,800	0.89 (0.69–1.16)	0.40	0.80 (0.57–1.14)	0.22	0.78 (0.55–1.10)	0.16	0.79 (0.56–1.11)	0.17
≥ 45,801	0.85 (0.63–1.13)	0.25	0.70 (0.48–1.02)	0.07	0.69 (0.47–0.99)	0.05	0.69 (0.47–0.99)	0.05
**Urbanisation**								
Level 1	1 (Reference)		1 (Reference)		1 (Reference)		1 (Reference)	
Level 2	1.17 (0.93–1.49)	0.18	0.97 (0.66–1.42)	0.86	1.14 (0.78–1.65)	0.50	0.94 (0.64–1.38)	0.76
Level 3	1.25 (0.96–1.65)	0.10	1.13 (0.73–1.73)	0.59	1.27 (0.83–1.94)	0.27	1.10 (0.72–1.68)	0.68
Level 4	1.53 (1.18–1.98)	< 0.01	0.78 (0.48–1.28)	0.33	1.14 (0.73–1.78)	0.57	0.86 (0.53–1.40)	0.55
**CCI at the year of index date**	1.36 (1.22–1.53)	< 0.01	1.31 (1.12–1.53)	< 0.01	1.32 (1.13–1.55)	< 0.01	1.32 (1.13–1.54)	< 0.01
**Extra-articular manifestations**								
Uveitis	3.00 (2.00–4.51)	< 0.01	1.53 (0.93–2.53)	0.09	1.48 (0.90–2.43)	0.13	1.49 (0.90–2.45)	0.12
Psoriasis	18.25 (9.08–36.66)	< 0.01	25.19 (9.52–66.61)	< 0.01	24.27 (9.25–63.69)	< 0.01	25.83 (9.74–68.53)	< 0.01
**Medications**								
NSAIDs	18.19 (14.01–23.62)	< 0.01	23.66 (8.96–62.49)	< 0.01	24.56 (9.28–65.03)	< 0.01	24.87 (9.36–66.06)	< 0.01
Methotrexate	12.90 (9.09–18.31)	< 0.01	4.50 (2.91–6.95)	< 0.01	4.42 (2.87–6.82)	< 0.01	4.42 (2.86–6.82)	< 0.01
Sulfasalazine	20.00 (13.46–29.71)	< 0.01	12.16 (8.98–16.46)	< 0.01	11.68 (8.66–15.76)	< 0.01	11.78 (8.73–15.90)	< 0.01
Corticosteroids, prednisolone equivalent dose (mg/day)	1.35 (1.26–1.46)	< 0.01	1.12 (1.06–1.18)	< 0.01	1.11 (1.05–1.18)	< 0.01	1.11 (1.05–1.18)	< 0.01
**Exposed levels of ambient air pollutants**								
PM2.5 (per 10 μg/m^3^)	0.94 (0.81–1.08)	0.37	0.88 (0.54–1.44)	0.61	0.74 (0.46–1.19)	0.21	0.77 (0.47–1.24)	0.28
PM10 (per 10 μg/m^3^)	0.99 (0.92–1.07)	0.78	1.07 (0.81–1.42)	0.61	1.16 (0.89–1.52)	0.28	1.15 (0.88–1.51)	0.30
SO_2_ (per 10 ppb)	0.32 (0.13–0.77)	0.01	0.80 (0.18–3.57)	0.78	0.23 (0.06–0.89)	0.03	0.38 (0.09–1.54)	0.18
NO_2_ (per 10 ppb)	0.73 (0.62–0.87)	< 0.01	0.23 (0.11–0.50)	< 0.01			0.59 (0.37–0.94)	0.03
CO (per 1 ppm)	0.62 (0.40–0.95)	0.03	8.57 (2.02–36.32)	< 0.01	0.93 (0.41–2.10)	0.86		
O_3_ (per 10 ppb)	1.34 (0.96–1.87)	0.08	0.51 (0.24–1.09)	0.08	1.03 (0.53–1.99)	0.93	0.60 (0.28–1.29)	0.19
**Akaike information criterion**			1675		1687		1682	
**Coefficient of determination**			0.32		0.32		0.32	

Models 2 and 3 are the same as models 2A and 3A in Supplementary Table 4, respectively. A *p*-value < 0.05 is considered statistically significant. *CCI* Charlson comorbidity index, *CI* Confidence interval, *NHI* National health insurance, *NSAIDs* Nonsteroidal anti-inflammatory drugs, *NTD* New Taiwan dollar, *PM* Particulate matter

^a^Model without adjustment for NO_2_ exposure

^b^Model without adjustment for CO exposure

As for exposed levels of air pollutants, the initiation of biologics was not associated with PM2.5, PM10 or SO_2_ levels. It tended to be negatively associated with O_3_ levels, whilst being positively associated with CO levels and negatively with NO_2_ levels in a significant manner. However, due to significant multicollinearity for NO_2_ and correlation between NO_2_ and CO levels, multivariable regression analyses without adjustment for NO_2_ and CO exposure were respectively presented as models 2 and 3. In both models, the difference from model 1 was significantly negative impact of higher monthly incomes (≥ 45,801 New Taiwan dollars). In model 2, the differences from model 1 were the negative impact of SO_2_ exposure and no impact of CO exposure. Otherwise, the results amongst the three models were consistent.

For the high correlation between PM10 and PM2.5 levels, multivariable regression models with adjustment for PM10 or PM2.5 exposure were presented in Supplementary Table 4, Additional file 1, showing consistent results regarding the adjustments. Models adjusting for PM10 or PM2.5 exposure using within 3 months before index date as the time horizon was demonstrated in Supplementary Table 5, Additional file 1, also showing consistent results amongst the models.

## Discussion

For adult patients with AS requiring medical therapy, the present study exhibited the positive association of active AS necessitating initiation of first NHI-reimbursed biologics with the disease duration, corresponding CCI, incidental psoriasis and use of NSAIDs, methotrexate, sulfasalazine and systemic corticosteroids in a dose-dependent manner within 1 year before biologics initiation. The main finding was that it was positively associated with exposed CO levels and negatively with NO_2_ levels, which had not yet been reported in the available English literature.

The novel finding was that exposure to ambient CO can be an aggravating factor, but exposure to ambient NO_2_ a protecting factor, regarding use of biologics indicated for high AS activity. Nevertheless, the impact of multicollinearity for NO_2_ and correlation between NO_2_ and CO levels might cause the variation of aORs for CO exposure amongst the models. Some studies reported a positive correlation between ambient CO and NO_2_ levels due to the influence of CO on the oxidation of NO to NO_2_, whilst both levels were found negatively correlated with O_3_ level as a result of photochemical reactions [18, 19]. These findings might explain the high correlation between CO and NO_2_ levels. Previous studies have abundantly elucidated the significant association between systemic inflammation and exposure to air pollutants [20, 21], yet a negative association with long-term O_3_ exposure from recent studies [22, 23]. Adequate use of ozone therapy has been introduced for patients with active AS despite insufficient explanations surrounding its immunological mechanism [24], but this may not be applicable to our finding of a potential protective effect seen in ambient O_3_. Although NO_2_ is a definite activator of airway inflammation [25], conflicting results were found regarding repeated daily exposure to 2 ppm of NO_2_ in small-sample human studies [26, 27]. Our results suggest a protective effect with outdoor NO_2_, which is conflicting with results of many studies but similar to that of a study investigating the influence of household NO_2_ [28]. Lacking available data presenting the impact of NO_2_ and O_3_ on AS, the abovementioned epidemiological and immunological gap deserves future research to clarify.

Air pollution was found to induce inflammatory immune responses, involving innate and adaptive immunity, from pulmonary to systemic sites [29, 30], and thus resulting in the development and progression of autoimmune diseases [31]. Nevertheless, the influence of air pollution on the pathogenesis and evolvement of AS has been insufficiently illustrated. Recent research has revealed the involvement of tumour necrosis factor and T helper 17 cells (Th17) in proinflammatory responses [32–34], which are the main components of immunopathological mechanisms in AS. Ambient PMs containing ligands of aryl hydrocarbon receptor, a cytosolic toxicant-responding transcription factor, enhance Th17 differentiation and may therefore potentiate underlying autoinflammation of AS [35, 36]. The relationship between air pollution and the response of pulmonary type 3 innate lymphoid cells has not yet been clearly investigated [37]. The immunological results were predominantly derived from cell-based studies, and whether these results can be translated into clinically significant immunopathology warrants additional in vivo studies to confirm.

We still found disease duration to be associated with AS activity after matching for duration within a range of 0.3 years, but we found low multicollinearity and correlation in terms of age at first NHI-reimbursed biologics initiation and disease duration (Supplementary Table 6, Additional file 1). As most of AS patients experience a disease course featuring intermittent flares [38], it is likely that AS patients exhibit evidence of high disease activity during follow-up visits coinciding with periods of disease flares. In Taiwan, many patients with low adherence to medications or ambulatory visits particularly seek medical attention only during disease flares and thus meet the NHI payment guidelines. This can lead to a bias towards a strong association. Disease activity was reported to be positively associated with corresponding comorbidities and medication use. Our results coincide with those in the majority of previous studies demonstrating that AS patients with comorbidities suffered from higher disease activity [39] and that those with active disease would confront higher medication and healthcare costs [40]. Patients with active AS in Taiwan would indeed have received regimens at a higher intensity to fulfil NHI payment guidelines for biologics administration. Although some studies perceived extra-articular manifestations as signs of uncontrolled systemic inflammation [41], we revealed that only incidental psoriasis within 1 year before biologics initiation had a strong association with high disease activity. According to an unadjusted comparison performed by Fitzgerald et al. [42], patients with comorbidities tended to have extra-articular manifestations, particularly psoriasis, compared with those having an isolated disease, whilst Zhao et al. did not report so [43]. This probably also reflected the differences in geographical and methodological backgrounds.

The available studies exploring the relationship between air pollution and spondyloarthritis are scarce. A case–control study reported a strong correlation between long-term PM2.5 exposure and worse AS outcomes [8]. Air pollution may also exacerbate joint symptoms in AS patients [7]. These findings, together with ours, indicate a negative impact of air pollution on AS evolvement. Park et al*.* revealed no association between long-term exposure to ambient PMs and incidence of AS in their cohort study [44], showing the limited pathogenetic role PMs play on AS. Facing these results, the impact of different study designs, recording or estimating methods of air pollutant levels, geographical, ethnic, cultural, political and economic issues should be considered for interpretation.

Knowing that air pollutants may modify inflammation, our study was the first to assess the respective association of the six ambient air pollutants with AS disease activity. As air pollution has become an issue attracting more concern worldwide, our study identified it as an urgency worthy of measures towards its prevention, monitoring and abatement for the benefit of public health. Our results may also provide a reference for environmental medicine specialists and administrative authorities to aid in the implementation of prompt policies. For rheumatologists and AS patients, such environmental risk factors surrounding disease deterioration deserve more attention to instructions on modifications to decrease exposure.

Our study has some strengths. First, use of a population-based database can minimise selection bias. Second, besides matching for gender, age at first NHI-reimbursed biologics initiation (± 3 years), year of AS diagnosis and disease duration (± 0.3 years), we adjusted many potential confounders, including socioeconomic status, comorbidities, extra-articular manifestations and use of medications for AS. Third, we conducted sensitivity analyses using different time horizons of air pollutants exposure. However, there are some limitations. First, the NHIRD lacked information on some potential confounding factors, including tobacco and alcohol use [45], dietary and exercise habits [8, 46] and stressful events [47]. Additionally, concomitant alternative medication use and real adherence to medications and outpatient follow-up remain indefinite, so chances are that subjects in the control group have high activity but biologics use. These are unmeasured potential confounders. Second, because of available claims data from 2003 to 2013 and estimated air pollutant levels from 2011, the time horizon of within 1 year before index date cannot speculate the influence from the longer-term exposure. However, we also conducted sensitivity analyses using the time horizon of 3 months and obtained consistent results. Third, although only patients having at least three ambulatory visits with AS diagnosis and concurrent AS-related medications are considered AS patients, the accuracy of AS diagnosis may still be of concern. Fourth, the estimated mean levels of exposed air pollutants at residential locations are unable to properly represent the exact amount of exposure in each subject. Fifth, the impact of the abovementioned multicollinearity is shown. Finally, the results of our study cannot be generalised to non-Tainanese populations. Future studies involving designs bearing higher potency evidence and longer follow-up periods, analysis models presenting the least influence of multicollinearity of air pollutants, studies using an individualised recording of exposed pollutant amounts and immunological research targeting the pathogenesis of AS remain warranted to confirm our findings.

## Conclusion

This nationwide, population-based study showed the initiation of NHI-reimbursed biologics, a proxy for high activity, in AS patients was positively associated with exposed ambient CO levels, but negatively associated with NO_2_ levels. The major limitations include the lack of information on individual smoking status and multicollinearity amongst levels of various air pollutants. Further studies are warranted to confirm our findings and elucidate the underlying mechanisms for the potential influence of ambient CO and NO_2_ on AS disease activity.

## Supplementary Information

**Additional file 1: Supplementary Table 1.** International Codes of Diseases–Ninth Revision Clinical Modification codes of diseases and manifestations, and Anatomical Therapeutic Chemical classification codes of medications. **Supplementary Table 2.** Baseline characteristics amongst matched study subjects with use of approved biologics through reimbursement and without the use. **Supplementary Table 3.** Correlation table for ambient air pollutant levels within three months before index date. **Supplementary Table 4.** Association between initiation of reimbursed biologics and air pollutants exposed within one year before index date in adjustment for potential confounders other than NO_2_ or CO exposure. **Supplementary Table 5.** Association between initiation of reimbursed biologics and air pollutants exposed within three months before index date in adjustment for potential confounders other than NO_2_ or CO exposure. **Supplementary Table 6.** Correlation table for age at first reimbursed biologic initiation and disease duration.

## Data Availability

The datasets used and/or analysed in the current study are available from the corresponding author upon reasonable request.

## Abbreviations

aOR: Adjusted odds ratio
AS: Ankylosing spondylitis
CCI: Charlson Comorbidity Index
CI: Confidence interval
CO: Carbon monoxide
ICD-9-CM: International Classification of Diseases-Ninth Revision-Clinical Modification
NHI: National Health Insurance
NHIRD: National Health Insurance Research Database
NO_2_: Nitrogen dioxide
NSAID: Non-steroidal anti-inflammatory drug
O_3_: Ozone
PM: Particulate matter
SO_2_: Sulphur dioxide
SPSS: Statistical Package for the Social Sciences
Th17: T helper 17 cells
VIF: Variance inflation factor

## References

[1] Merino M, Brace O, Gonzalez-Dominguez A, Hidalgo-Vega A, Garrido-Cumbrera M, Gratacos J (2021). Social economic costs of ankylosing spondylitis in Spain. Clin Exper Rheumatol.

[2] Sunkureddi P, Gibson D, Doogan S, Heid J, Benosman S, Park Y (2018). Using self-reported patient experiences to understand patient burden: learnings from digital patient communities in ankylosing spondylitis. Adv Ther.

[3] Hsieh M-Y, Kuo C-F (2016). FRI0428 Epidemiology of ankylosing spondylitis in Taiwan: a nationwide population study. Ann Rheum Dis.

[4] Ramiro S, Nikiphorou E, Sepriano A, Ortolan A, Webers C, Baraliakos X (2023). ASAS-EULAR recommendations for the management of axial spondyloarthritis: 2022 update. Ann Rheum Dis.

[5] Hiraiwa K, van Eeden SF (2013). Contribution of lung macrophages to the inflammatory responses induced by exposure to air pollutants. Mediators Inflamm.

[6] Adami G, Pontalti M, Cattani G, Rossini M, Viapiana O, Orsolini G (2022). Association between long-term exposure to air pollution and immune-mediated diseases: a population-based cohort study. RMD Open..

[7] Ziade N, Bouzamel M, Mrad-Nakhle M, Abi Karam G, Hmamouchi I, Abouqal R (2021). Prospective correlational time-series analysis of the influence of weather and air pollution on joint pain in chronic rheumatic diseases. Clin Rheumatol.

[8] Soleimanifar N, Nicknam MH, Bidad K, Jamshidi AR, Mahmoudi M, Mostafaei S (2019). Effect of food intake and ambient air pollution exposure on ankylosing spondylitis disease activity. Adv Rheumatol.

[9] Cheng TM (2003). Taiwan's new national health insurance program: genesis and experience so far. Health Aff (Millwood).

[10] Qi Y, Li Q, Karimian H, Liu D (2019). A hybrid model for spatiotemporal forecasting of PM2.5 based on graph convolutional neural network and long short-term memory. Sci Total Environ.

[11] Taiwan Air Quality Monitoring Network. 2021. https://airtw.epa.gov.tw/ENG/default.aspx. Accessed 2 Mar 2021.

[12] van der Linden S, Valkenburg HA, Cats A (1984). Evaluation of diagnostic criteria for ankylosing spondylitis. A proposal for modification of the New York criteria. Arthritis Rheum..

[13] Rudwaleit M, Landewe R, van der Heijde D, Listing J, Brandt J, Braun J (2009). The development of Assessment of SpondyloArthritis international Society classification criteria for axial spondyloarthritis (part I): classification of paper patients by expert opinion including uncertainty appraisal. Ann Rheum Dis.

[14] Rudwaleit M, van der Heijde D, Landewe R, Listing J, Akkoc N, Brandt J (2009). The development of Assessment of SpondyloArthritis international Society classification criteria for axial spondyloarthritis (part II): validation and final selection. Ann Rheum Dis.

[15] Grimes DA, Schulz KF (2005). Compared to what? Finding controls for case-control studies. Lancet.

[16] Chen BY, Chen CH, Chuang YC, Wu YH, Pan SC, Guo YL (2019). Changes in the relationship between childhood asthma and ambient air pollution in Taiwan: Results from a nationwide survey repeated 5 years apart. Pediatr Allergy Immunol.

[17] Deyo RA, Cherkin DC, Ciol MA (1992). Adapting a clinical comorbidity index for use with ICD-9-CM administrative databases. J Clin Epidemiol.

[18] Kovač-Andrić E, Radanović T, Topalović I, Marković B, Sakač N (2013). Temporal variations in concentrations of ozone, nitrogen dioxide, and carbon monoxide at Osijek. Croatia Adv Meteorol.

[19] Butenhoff CL, Khalil MA, Porter WC, Al-Sahafi MS, Almazroui M, Al-Khalaf A (2015). Evaluation of ozone, nitrogen dioxide, and carbon monoxide at nine sites in Saudi Arabia during 2007. J Air Waste Manag Assoc.

[20] Liu Q, Gu X, Deng F, Mu L, Baccarelli AA, Guo X (2019). Ambient particulate air pollution and circulating C-reactive protein level: a systematic review and meta-analysis. Int J Hyg Environ Health.

[21] Xu Z, Wang W, Liu Q, Li Z, Lei L, Ren L (2022). Association between gaseous air pollutants and biomarkers of systemic inflammation: a systematic review and meta-analysis. Environ Pollut.

[22] Liu K, Cao H, Li B, Guo C, Zhao W, Han X (2022). Long-term exposure to ambient nitrogen dioxide and ozone modifies systematic low-grade inflammation: The CHCN-BTH study. Int J Hyg Environ Health.

[23] Kim JH, Woo HD, Choi S, Song DS, Lee JH, Lee K (2022). Long-term effects of ambient particulate and gaseous pollutants on serum high-sensitivity C-reactive protein levels: a cross-sectional study using KoGES-HEXA data. Int J Environ Res Public Health.

[24] Inci H, İnci F (2023). Effect of ozone therapy on neutrophil/lymphocyte, platelet/lymphocyte ratios, and disease activity in ankylosing spondylitis: a self-controlled randomized study. Med Gas Res.

[25] Ji X, Han M, Yun Y, Li G, Sang N (2015). Acute nitrogen dioxide (NO2) exposure enhances airway inflammation via modulating Th1/Th2 differentiation and activating JAK-STAT pathway. Chemosphere.

[26] Blomberg A, Krishna MT, Helleday R, Soderberg M, Ledin MC, Kelly FJ (1999). Persistent airway inflammation but accommodated antioxidant and lung function responses after repeated daily exposure to nitrogen dioxide. Am J Respir Crit Care Med.

[27] Pathmanathan S, Krishna MT, Blomberg A, Helleday R, Kelly FJ, Sandstrom T (2003). Repeated daily exposure to 2 ppm nitrogen dioxide upregulates the expression of IL-5, IL-10, IL-13, and ICAM-1 in the bronchial epithelium of healthy human airways. Occup Environ Med.

[28] Fandino-Del-Rio M, Kephart JL, Williams KN, Malpartida G, Boyd Barr D, Steenland K (2021). Household air pollution and blood markers of inflammation: a cross-sectional analysis. Indoor Air.

[29] Zhao CN, Xu Z, Wu GC, Mao YM, Liu LN, Qian W (2019). Emerging role of air pollution in autoimmune diseases. Autoimmun Rev.

[30] Glencross DA, Ho TR, Camina N, Hawrylowicz CM, Pfeffer PE (2020). Air pollution and its effects on the immune system. Free Radic Biol Med.

[31] Gawda A, Majka G, Nowak B, Marcinkiewicz J (2017). Air pollution, oxidative stress, and exacerbation of autoimmune diseases. Cent Eur J Immunol.

[32] Cox FA, Stiller-Winkler R, Hadnagy W, Ranft U, Idel H (1999). Soluble tumor necrosis factor receptor (sTNF RII) in sera of children and traffic-derived particulate air pollution. Zentralbl Hyg Umweltmed.

[33] Nakamura R, Inoue K, Fujitani Y, Kiyono M, Hirano S, Takano H (2012). Effects of nanoparticle-rich diesel exhaust particles on IL-17 production in vitro. J Immunotoxicol.

[34] Mann EH, Ho TR, Pfeffer PE, Matthews NC, Chevretton E, Mudway I (2017). Vitamin D counteracts an IL-23-dependent IL-17A(+)IFN-gamma(+) response driven by urban particulate matter. Am J Respir Cell Mol Biol.

[35] Veldhoen M, Hirota K, Westendorf AM, Buer J, Dumoutier L, Renauld JC (2008). The aryl hydrocarbon receptor links TH17-cell-mediated autoimmunity to environmental toxins. Nature.

[36] van Voorhis M, Knopp S, Julliard W, Fechner JH, Zhang X, Schauer JJ (2013). Exposure to atmospheric particulate matter enhances Th17 polarization through the aryl hydrocarbon receptor. PLoS ONE.

[37] Estrella B, Naumova EN, Cepeda M, Voortman T, Katsikis PD, Drexhage HA (2019). Effects of air pollution on lung innate lymphoid cells: review of in vitro and in vivo experimental studies. Int J Environ Res Public Health.

[38] Stone MA, Pomeroy E, Keat A, Sengupta R, Hickey S, Dieppe P (2008). Assessment of the impact of flares in ankylosing spondylitis disease activity using the Flare Illustration. Rheumatology (Oxford).

[39] Zhao SS, Robertson S, Reich T, Harrison NL, Moots RJ, Goodson NJ (2020). Prevalence and impact of comorbidities in axial spondyloarthritis: systematic review and meta-analysis. Rheumatology (Oxford).

[40] Ara RM, Packham JC, Haywood KL (2008). The direct healthcare costs associated with ankylosing spondylitis patients attending a UK secondary care rheumatology unit. Rheumatology (Oxford).

[41] Elewaut D, Matucci-Cerinic M (2009). Treatment of ankylosing spondylitis and extra-articular manifestations in everyday rheumatology practice. Rheumatology (Oxford).

[42] Fitzgerald G, Gallagher P, O'Shea FD (2020). Multimorbidity in axial spondyloarthropathy and its association with disease outcomes: results from the Ankylosing Spondylitis Registry of Ireland cohort. J Rheumatol.

[43] Zhao SS, Radner H, Siebert S, Duffield SJ, Thong D, Hughes DM (2019). Comorbidity burden in axial spondyloarthritis: a cluster analysis. Rheumatology (Oxford).

[44] Park JS, Choi S, Kim K, Chang J, Kim SM, Kim SR (2021). Association of particulate matter with autoimmune rheumatic diseases among adults in South Korea. Rheumatology (Oxford).

[45] Wieczorek M, Gwinnutt JM, Ransay-Colle M, Balanescu A, Bischoff-Ferrari H, Boonen A (2022). Smoking, alcohol consumption and disease-specific outcomes in rheumatic and musculoskeletal diseases (RMDs): systematic reviews informing the 2021 EULAR recommendations for lifestyle improvements in people with RMDs. RMD Open.

[46] Harpham C, Harpham QK, Barker AR (2022). The effect of exercise training programs with aerobic components on C-reactive protein, erythrocyte sedimentation rate and self-assessed disease activity in people with ankylosing spondylitis: a systematic review and meta-analysis. Int J Rheum Dis.

[47] Zeboulon-Ktorza N, Boelle PY, Nahal RS, D'Agostino MA, Vibert JF, Turbelin C (2013). Influence of environmental factors on disease activity in spondyloarthritis: a prospective cohort study. J Rheumatol.

