# Supplementary material for: Association between air pollutants and initiation of biological therapy in patients with ankylosing spondylitis: a nationwide, population-based, nested case–control study
Source: Arthritis Res Ther. 2023 May 5;25:75. doi: 10.1186/s13075-023-03060-4 (PMC10161550; doi:10.1186/s13075-023-03060-4)
Supplement: Supplementary file 1 — Additional file 1: Supplementary Table 1. International Codes of Diseases–Ninth Revision Clinical Modification codes of diseases and manifestations, and Anatomical Therapeutic Chemical classification codes of medications. Supplementary Table 2. Baseline characteristics amongst matched study subjects with use of approved biologics through reimbursement and without the use. Supplementary Table 3. Correlation table for ambient air pollutant levels within three months before index date. Supplementary Table 4. Association between initiation of reimbursed biologics and air pollutants exposed within one year before index date in adjustment for potential confounders other than NO2 or CO exposure. Supplementary Table 5. Association between initiation of reimbursed biologics and air pollutants exposed within three months before index date in adjustment for potential confounders other than NO2 or CO exposure. Supplementary Table 6. Correlation table for age at first reimbursed biologic initiation and disease duration. [file 13075_2023_3060_MOESM1_ESM.docx]

**Supplementary Table 1. International Codes of Diseases–Ninth Revision Clinical Modification codes of diseases and manifestations, and Anatomical Therapeutic Chemical classification codes of medications**

| Items | Codes |
| --- | --- |
| **Rheumatic diseases** |  |
| Ankylosing spondylitis | 720.0 |
| Rheumatoid arthritis | 714.0 |
| **Component diagnoses of Charlson Comorbidity Index** |  |
| Acquired immunodeficiency syndrome | 042-044 |
| Cerebrovascular disease | 430-438 |
| Chronic obstructive pulmonary disease | 500-505,506.4 |
| Congestive heart failure | 428 |
| Connective tissue diseases | 710.0, 710.1, 710.4, 714.0, 714.1, 714.2, 714.81, 725 |
| Dementia | 290 |
| Diabetes mellitus | 250.0-250.3, 250.7 |
| Diabetes mellitus with end-organ damage | 250.4-250.6 |
| Hemiplegia | 344.1 |
| Metastatic solid tumors | 196-199 |
| Mild liver diseases | 571.2, 571.4-571.6 |
| Moderate or severe liver diseases | 456.0-456.2, 572.2-572.8 |
| Moderate to severe renal diseases | 582, 583.0-583.7, 585, 586, 588 |
| Myocardial infarction | 410, 412 |
| Peptic ulcer disease | 531-534 |
| Peripheral vascular disease | 443.9, 441, 785.4, V43.4 |
| Tumours | 140-195, 200-208 |
| **Extra-articular manifestations** |  |
| Uveitis | 364.00–364.02, 364.04–364.05, 364.3 |
| Psoriasis | 696.1 |
| Inflammatory bowel disease | 555-556 |
| **Medications** |  |
| NSAIDs | M01A |
| Methotrexate | L04AX03 |
| Sulfasalazine | A07EC01 |
| Dexamethasone | H02AB02 |
| Hydrocortisone | H02AB09 |
| Methylprednisolone | H02AB04 |
| Prednisolone | H02AB06 |
| Adalimumab | L04AB04 |
| Etanercept | L04AB01 |
| Golimumab | L04AB06 |

**Supplementary Table 2. Baseline characteristics amongst matched study subjects with use of approved biologics through reimbursement and without the use.**

|  | **Controls**  **(*n* = 2336)** | **Etanercept**  **(*n* = 237)** | **Adalimumab**  **(*n* = 242)** | **Golimumab**  **(*n* = 105)** | ***p-value*** |
| --- | --- | --- | --- | --- | --- |
| **Gender** |  |  |  |  |  |
| Female, *n* (%) | 372 (15.9) | 44 (18.6) | 38 (15.7) | 11 (10.5) | 0.31 |
| Male, *n* (%) | 1964 (84.1) | 193 (81.4) | 204 (84.3) | 94 (89.5) |  |
| **Age of first NHI-reimbursed biologic initiation**, mean ± S.D. | 40.4 ± 12.5 | 40.5 ± 13.3 | 40.0 ± 12.2 | 40.0 ± 13.0 | 0.96 |
| **Disease duration (year)**, mean ± S.D. | 6.0 ± 3.5 | 5.7 ± 3.7 | 6.2 ± 3.3 | 6.8 ± 3.6 | 0.05 |
| **Monthly income (NTD)** |  |  |  |  | 0.43 |
| ≤ 15,840, *n* (%) | 656 (28.1) | 83 (35.0) | 63 (26.0) | 30 (28.6) |  |
| 15,841–28,800, *n* (%) | 812 (34.8) | 79 (33.3) | 86 (35.5) | 39 (37.1) |  |
| 28,801–45,800, *n* (%) | 478 (20.5) | 37 (15.6) | 56 (23.1) | 22 (21.0) |  |
| ≥ 45,801, *n* (%) | 390 (16.7) | 38 (16.0) | 37 (15.3) | 14 (13.3) |  |
| **Urbanisation** |  |  |  |  | 0.09 |
| Level 1, *n* (%) | 757 (32.4) | 63 (26.6) | 62 (25.6) | 33 (31.4) |  |
| Level 2, *n* (%) | 738 (31.6) | 74 (31.2) | 74 (30.6) | 33 (31.4) |  |
| Level 3, *n* (%) | 416 (17.8) | 48 (20.3) | 48 (19.8) | 13 (12.4) |  |
| Level 4, *n* (%) | 425 (18.2) | 52 (21.9) | 58 (24.0) | 26 (24.8) |  |
| **CCI at the year of index date,** mean ± S.D. | 0.2 ± 0.7 | 0.5 ± 0.9 | 0.4 ± 0.7 | 0.4 ± 0.6 | < 0.01 |
| **CCI subgroups** |  |  |  |  | < 0.01 |
| CCI = 0, *n* (%) | 1994 (85.4) | 163 (68.8) | 168 (69.4) | 74 (70.5) |  |
| CCI ≥ 1, *n* (%) | 342 (14.6) | 74 (31.2) | 74 (30.6) | 31 (29.5) |  |
| **Extra-articular manifestations** |  |  |  |  |  |
| Uveitis, *n* (%) | 59 (2.5) | 14 (5.9) | 18 (7.4) | 10 (9.5) | < 0.01 |
| Psoriasis, *n* (%) | 10 (0.4) | 24 (10.1) | 14 (5.8) | 4 (3.8) | < 0.01 |
| Inflammatory bowel disease, *n* (%) | 7 (0.3) | 1 (0.4) | 6 (2.5) | 0 (0.0) | < 0.01 |
| **Medications** |  |  |  |  |  |
| NSAIDs, *n* (%) | 1663 (71.2) | 234 (98.7) | 240 (99.2) | 105 (100.0) | < 0.01 |
| Methotrexate, *n* (%) | 50 (2.1) | 60 (25.3) | 47 (19.4) | 17 (16.2) | < 0.01 |
| Sulfasalazine, *n* (%) | 656 (28.1) | 202 (85.2) | 210 (86.8) | 94 (89.5) | < 0.01 |
| Corticosteroids, *n* (%) | 492 (21.1) | 119 (50.2) | 112 (46.3) | 61 (58.1) | < 0.01 |
| Corticosteroids, prednisolone equivalent dose (mg/day), mean ± S.D. | 0.2 ± 1.9 | 1.1 ± 2.2 | 1.3 ± 3.5 | 1.2 ± 2.2 | < 0.01 |
| **Exposed levels of ambient air pollutants** |  |  |  |  |  |
| PM2.5 (per 10 µg/m^3^), mean ± S.D. | 3.0 ± 0.6 | 3.0±0.6 | 2.9±0.6 | 3.1±0.6 | 0.39 |
| PM10 (per 10 µg/m^3^), mean ± S.D. | 5.2 ± 1.2 | 5.2±1.2 | 5.1±1.1 | 5.3±1.2 | 0.42 |
| SO_2_ (per 10 ppb), mean ± S.D. | 0.4 ± 0.1 | 0.4±0.1 | 0.3±0.1 | 0.4±0.1 | 0.01 |
| NO_2_ (per 10 ppb), mean ± S.D. | 1.9 ± 0.6 | 1.8±0.5 | 1.8±0.5 | 1.8±0.5 | < 0.01 |
| CO (per 1 ppm), mean ± S.D. | 0.6 ± 0.2 | 0.5±0.2 | 0.5±0.2 | 0.5±0.2 | 0.15 |
| O_3_ (per 10ppb), mean ± S.D. | 2.8 ± 0.3 | 2.8±0.3 | 2.8±0.3 | 2.8±0.3 | 0.28 |

A *p*-value < 0.05 is considered statistically significant. CCI, Charlson comorbidity index. NHI, national health insurance. NSAIDs, nonsteroidal anti-inflammatory drugs. NTD, New Taiwan dollar. PM, particulate matter. S.D., standard deviation.

**Supplementary Table 3. Correlation table for ambient air pollutant levels within three months before index date.**

| **(*n* = 2,920)** | **VIF** | PM2.5  (per 10 µg/m^3^) | PM10  (per 10 µg/m^3^) | SO_2_  (per 10 ppb) | NO_2_  (per 10 ppb) | CO  (per 1 ppm) | O_3_  (per 10ppb) |
| --- | --- | --- | --- | --- | --- | --- | --- |
| PM2.5 (per 10 µg/m^3^) | 8 | 1 | 0.932 | 0.473 | 0.202 | 0.079 | 0.307 |
| PM10 (per 10 µg/m^3^) | 9 | < 0.01 | 1 | 0.497 | 0.127 | 0.034 | 0.378 |
| SO_2_ (per 10 ppb) | 2 | < 0.01 | < 0.01 | 1 | 0.258 | 0.093 | 0.007 |
| NO_2_ (per 10 ppb) | 8 | < 0.01 | < 0.01 | < 0.01 | 1 | 0.900 | -0.760 |
| CO (per 1 ppm) | 6 | < 0.01 | < 0.01 | < 0.01 | < 0.01 | 1 | -0.670 |
| O_3_ (per 10ppb) | 2 | < 0.01 | < 0.01 | < 0.01 | < 0.01 | < 0.01 | 1 |

The VIF of each air pollutant indicates the level of multicollinearity with others, and VIF ≥ 10 is considered significant multicollinearity necessitating being corrected. For each pair of air pollutants, Pearson’s correlation coefficient is presented from the right upper part, and the *p*-value from the left lower part of the table. A *p*-value < 0.05 is considered statistically significant. PM, particulate matter. VIF, variance inflation factor.

**Supplementary Table 4. Association between initiation of reimbursed biologics and air pollutants exposed within one year before index date in adjustment for potential confounders other than NO_2_ or CO exposure.**

|  | **Multivariable analyses without adjustment for NO_2_ exposure** | | | | | | **Multivariable analyses without adjustment for CO exposure** | | | | | |
| --- | --- | --- | --- | --- | --- | --- | --- | --- | --- | --- | --- | --- |
|  | **Model 2A** | | **Model 2B *** | | **Model 2C #** | | **Model 3A** | | **Model 3B *** | | **Model 3C #** | |
|  | **Adjusted OR**  **(95% CI)** | ***p*-value** | **Adjusted OR**  **(95% CI)** | ***p*-value** | **Adjusted OR**  **(95% CI)** | ***p*-value** | **Adjusted OR**  **(95% CI)** | ***p*-value** | **Adjusted OR**  **(95% CI)** | ***p*-value** | **Adjusted OR**  **(95% CI)** | ***p*-value** |
| **Age at first NHI-reimbursed biologic initiation** | 1.00  (0.99–1.01) | 0.77 | 1.00  (0.99–1.01) | 0.76 | 1.00  (0.99–1.01) | 0.77 | 1.00  (0.99–1.01) | 0.82 | 1.00  (0.99–1.01) | 0.80 | 1.00  (0.99–1.01) | 0.82 |
| **Disease duration** | 9.25  (6.18–13.86) | < 0.01 | 9.21  (6.15–13.80) | < 0.01 | 9.24  (6.17–13.84) | < 0.01 | 9.19  (6.14–13.77) | < 0.01 | 9.16  (6.11-13.71) | < 0.01 | 9.18  (6.13–13.75) | < 0.01 |
| **Monthly income (NTD)** |  |  |  |  |  |  |  |  |  |  |  |  |
| ≤ 15,840 | 1 (Reference) |  | 1 (Reference) |  | 1 (Reference) |  | 1 (Reference) |  | 1 (Reference) |  | 1 (Reference) |  |
| 15,841–28,800 | 0.81  (0.60–1.09) | 0.16 | 0.80  (0.59–1.08) | 0.15 | 0.80  (0.59–1.08) | 0.14 | 0.80  (0.59–1.08) | 0.14 | 0.79  (0.59–1.07) | 0.13 | 0.79  (0.58–1.07) | 0.12 |
| 28,801–45,800 | 0.78  (0.55–1.10) | 0.16 | 0.78  (0.55–1.10) | 0.16 | 0.78  (0.55–1.11) | 0.16 | 0.79  (0.56–1.11) | 0.17 | 0.79  (0.56–1.11) | 0.17 | 0.79  (0.56–1.11) | 0.18 |
| ≥ 45,801 | 0.69  (0.47–0.99) | 0.05 | 0.68  (0.47–0.98) | 0.04 | 0.68  (0.47–0.99) | 0.04 | 0.69  (0.47–0.99) | 0.05 | 0.68  (0.47–0.99) | 0.04 | 0.68  (0.47–0.99) | 0.05 |
| **Urbanisation** |  |  |  |  |  |  |  |  |  |  |  |  |
| Level 1 | 1 (Reference) |  | 1 (Reference) |  | 1 (Reference) |  | 1 (Reference) |  | 1 (Reference) |  | 1 (Reference) |  |
| Level 2 | 1.14  (0.78–1.65) | 0.50 | 1.16  (0.80–1.68) | 0.43 | 1.18  (0.82–1.70) | 0.39 | 0.94  (0.64–1.38) | 0.76 | 0.95  (0.65–1.39) | 0.79 | 0.96  (0.66–1.40) | 0.83 |
| Level 3 | 1.27  (0.83–1.94) | 0.27 | 1.29  (0.85–1.97) | 0.23 | 1.30  (0.85–1.98) | 0.22 | 1.10  (0.72–1.68) | 0.68 | 1.11  (0.72–1.69) | 0.64 | 1.11  (0.73–1.69) | 0.63 |
| Level 4 | 1.14  (0.73–1.78) | 0.57 | 1.20  (0.77–1.86) | 0.42 | 1.19  (0.77–1.86) | 0.44 | 0.86  (0.53–1.40) | 0.55 | 0.89  (0.55–1.45) | 0.65 | 0.88  (0.54–1.43) | 0.61 |
| **CCI at the year of index date** | 1.32  (1.13–1.55) | < 0.01 | 1.33  (1.13–1.55) | < 0.01 | 1.32  (1.13–1.54) | < 0.01 | 1.32  (1.13–1.54) | < 0.01 | 1.32  (1.13–1.54) | < 0.01 | 1.32  (1.13–1.54) | < 0.01 |
| **Extra-articular manifestations** |  |  |  |  |  |  |  |  |  |  |  |  |
| Uveitis | 1.48  (0.90–2.43) | 0.13 | 1.47  (0.89–2.42) | 0.14 | 1.46  (0.89–2.41) | 0.14 | 1.49  (0.90–2.45) | 0.12 | 1.48  (0.90–2.43) | 0.13 | 1.48  (0.90–2.43) | 0.133 |
| Psoriasis | 24.27  (9.25–63.69) | < 0.01 | 24.51  (9.34–64.36) | < 0.01 | 24.39  (9.29–64.03) | < 0.01 | 25.83  (9.74–68.53) | < 0.01 | 26.15  (9.85–69.45) | < 0.01 | 26.02  (9.80–69.13) | < 0.01 |
| **Medications** |  |  |  |  |  |  |  |  |  |  |  |  |
| NSAIDs | 24.56  (9.28–65.03) | < 0.01 | 24.45  (9.25–64.61) | < 0.01 | 24.45  (9.25–64.68) | < 0.01 | 24.87  (9.36–66.06) | < 0.01 | 24.80  (9.35–65.77) | < 0.01 | 24.80  (9.34–65.85) | < 0.01 |
| Methotrexate | 4.42  (2.87–6.82) | < 0.01 | 4.41  (2.86–6.80) | < 0.01 | 4.45  (2.88–6.85) | < 0.01 | 4.42  (2.86–6.82) | < 0.01 | 4.41  (2.86–6.80) | < 0.01 | 4.44  (2.88–6.85) | < 0.01 |
| Sulfasalazine | 11.68  (8.66–15.76) | < 0.01 | 11.67  (8.65–15.75) | < 0.01 | 11.65  (8.64–15.71) | < 0.01 | 11.78  (8.73–15.90) | < 0.01 | 11.77  (8.72–15.88) | < 0.01 | 11.75  (8.71–15.85) | < 0.01 |
| Corticosteroids, prednisolone equivalent dose (mg/day) | 1.11  (1.05–1.18) | < 0.01 | 1.11  (1.05–1.18) | < 0.01 | 1.11  (1.05–1.17) | < 0.01 | 1.11  (1.05–1.18) | < 0.01 | 1.11  (1.05–1.18) | < 0.01 | 1.11  (1.05–1.18) | < 0.01 |
| **Exposed levels of ambient air pollutants** |  |  |  |  |  |  |  |  |  |  |  |  |
| PM2.5 (per 10 µg/m^3^) | 0.74  (0.46–1.19) | 0.21 | 0.93  (0.74–1.17) | 0.52 |  |  | 0.77  (0.47–1.24) | 0.28 | 0.96  (0.76–1.20) | 0.70 |  |  |
| PM10 (per 10 µg/m^3^) | 1.16  (0.89–1.52) | 0.28 |  |  | 1.00  (0.87–1.14) | 0.96 | 1.15  (0.88–1.51) | 0.30 |  |  | 1.01  (0.89–1.15) | 0.88 |
| SO_2_ (per 10 ppb) | 0.23  (0.06–0.89) | 0.03 | 0.29  (0.08–1.03) | 0.06 | 0.24  (0.06–0.91) | 0.04 | 0.38  (0.09–1.54) | 0.18 | 0.46  (0.12–1.78) | 0.26 | 0.40  (0.10–1.61) | 0.20 |
| NO_2_ (per 10 ppb) |  |  |  |  |  |  | 0.59  (0.37–0.94) | 0.03 | 0.59  (0.37–0.94) | 0.03 | 0.58  (0.37–0.93) | 0.02 |
| CO (per 1 ppm) | 0.93  (0.41–2.10) | 0.86 | 0.97  (0.43–2.18) | 0.94 | 0.96  (0.42–2.16) | 0.92 |  |  |  |  |  |  |
| O_3_ (per 10ppb) | 1.03  (0.53–1.99) | 0.93 | 1.12  (0.59–2.12) | 0.74 | 1.08  (0.56–2.07) | 0.83 | 0.60  (0.28–1.29) | 0.19 | 0.64  (0.30–1.35) | 0.24 | 0.61  (0.28–1.30) | 0.20 |
| **Akaike information criterion** | 1,687 | | 1,686 | | 1,686 | | 1,682 | | 1,681 | | 1,681 | |
| **Coefficient of determination** | 0.32 | | 0.32 | | 0.32 | | 0.32 | | 0.32 | | 0.32 | |

Models 2A and 3A are the same as models 2 and 3 in Table 3, respectively. A *p*-value < 0.05 is considered statistically significant. Adjusted OR, adjusted odds ratio. CCI, Charlson comorbidity index. CI, confidence interval. NHI, national health insurance. NSAIDs, nonsteroidal anti-inflammatory drugs. NTD, New Taiwan dollar. PM, particulate matter.

***** Models without adjustment for PM10 exposure.

**#** Models without adjustment for PM2.5 exposure.

**Supplementary Table 5. Association between initiation of reimbursed biologics and air pollutants exposed within three months before index date in adjustment for potential confounders other than NO_2_ or CO exposure.**

|  | **Multivariable analyses without adjustment for NO_2_ exposure** | | | | **Multivariable analyses without adjustment for CO exposure** | | | | |
| --- | --- | --- | --- | --- | --- | --- | --- | --- | --- |
|  | **Model 2A *** | | **Model 2B #** | | **Model 3A *** | | **Model 3B #** | | |
|  | **Adjusted OR**  **(95% CI)** | ***p*-value** | **Adjusted OR**  **(95% CI)** | ***p*-value** | **Adjusted OR**  **(95% CI)** | ***p*-value** | **Adjusted OR**  **(95% CI)** | ***p*-value** |  |
| **Age at first NHI-reimbursed biologic initiation** | 1.00  (0.99–1.01) | 0.76 | 1.00  (0.99–1.01) | 0.76 | 1.00  (0.99–1.01) | 0.73 | 1.00  (0.99–1.01) | 0.73 |  |
| **Disease duration** | 7.39  (4.91–11.12) | < 0.01 | 7.42  (4.93–11.18) | < 0.01 | 6.71  (4.44-10.14) | < 0.01 | 6.75  (4.47–10.20) | < 0.01 |  |
| **Monthly income (NTD)** |  |  |  |  |  |  |  |  |  |
| ≤ 15,840 | 1 (Reference) |  | 1 (Reference) |  | 1 (Reference) |  | 1 (Reference) |  |  |
| 15,841–28,800 | 0.79  (0.59–1.08) | 0.14 | 0.79  (0.58–1.08) | 0.14 | 0.77  (0.57–1.05) | 0.10 | 0.77  (0.57–1.05) | 0.10 |  |
| 28,801–45,800 | 0.82  (0.57–1.16) | 0.26 | 0.82  (0.57–1.16) | 0.26 | 0.83  (0.59–1.19) | 0.32 | 0.83  (0.59–1.19) | 0.32 |  |
| ≥ 45,801 | 0.68  (0.46–0.98) | 0.04 | 0.68  (0.46–0.98) | 0.04 | 0.70  (0.48–1.02) | 0.06 | 0.70  (0.48–1.03) | 0.07 |  |
| **Urbanisation** |  |  |  |  |  |  |  |  |  |
| Level 1 | 1 (Reference) |  | 1 (Reference) |  | 1 (Reference) |  | 1 (Reference) |  |  |
| Level 2 | 1.50  (1.04–2.17) | 0.03 | 1.49  (1.03–2.16) | 0.03 | 1.16  (0.80–1.69) | 0.43 | 1.16  (0.80–1.69) | 0.44 |  |
| Level 3 | 1.90  (1.25–2.89) | < 0.01 | 1.89  (1.24–2.87) | < 0.01 | 1.49  (0.98–2.28) | 0.06 | 1.49  (0.98–2.28) | 0.06 |  |
| Level 4 | 1.95  (1.26–3.02) | < 0.01 | 1.92  (1.23–2.99) | < 0.01 | 1.25  (0.77–2.01) | 0.37 | 1.23  (0.76–1.99) | 0.41 |  |
| **CCI at the year of index date** | 1.39  (1.18–1.64) | < 0.01 | 1.39  (1.18–1.64) | < 0.01 | 1.37  (1.17–1.62) | < 0.01 | 1.37  (1.17–1.62) | < 0.01 |  |
| **Extra-articular manifestations** |  |  |  |  |  |  |  |  |  |
| Uveitis | 1.67  (1.01–2.76) | 0.05 | 1.67  (1.01–2.77) | 0.05 | 1.71  (1.04–2.83) | 0.04 | 1.72  (1.04–2.84) | 0.04 |  |
| Psoriasis | 27.58  (10.20–74.53) | < 0.01 | 27.42  (10.15–74.12) | < 0.01 | 30.17  (10.94–83.21) | < 0.01 | 29.84  (10.83–82.23) | < 0.01 |  |
| **Medications** |  |  |  |  |  |  |  |  |  |
| NSAIDs | 26.57  (10.00–70.58) | < 0.01 | 26.62  (10.01–70.76) | < 0.01 | 27.46  (10.30–73.25) | < 0.01 | 27.48  (10.31–73.29) | < 0.01 |  |
| Methotrexate | 4.34  (2.80–6.73) | < 0.01 | 4.35  (2.81–6.75) | < 0.01 | 4.50  (2.90–6.99) | < 0.01 | 4.49  (2.89–6.97) | < 0.01 |  |
| Sulfasalazine | 11.90  (8.79–16.11) | < 0.01 | 11.89  (8.78–16.10) | < 0.01 | 12.14  (8.95–16.47) | < 0.01 | 12.12  (8.93–16.44) | < 0.01 |  |
| Corticosteroids, prednisolone equivalent dose (mg/day) | 1.11  (1.04–1.17) | < 0.01 | 1.11  (1.04–1.17) | < 0.01 | 1.11  (1.05–1.18) | < 0.01 | 1.11  (1.05–1.18) | < 0.01 |  |
| **Exposed levels of ambient air pollutants** |  |  |  |  |  |  |  |  |  |
| PM2.5 (per 10 µg/m^3^) | 1.01  (0.84–1.21) | 0.95 |  |  | 1.15  (0.95–1.39) | 0.17 |  |  |  |
| PM10 (per 10 µg/m^3^) |  |  | 1.02  (0.92–1.13) | 0.74 |  |  | 1.08  (0.97–1.21) | 0.15 |  |
| SO_2_ (per 10 ppb) | 0.65  (0.18–2.35) | 0.52 | 0.60  (0.16–2.23) | 0.45 | 1.28  (0.34–4.82) | 0.71 | 1.19  (0.31–4.60) | 0.80 |  |
| NO_2_ (per 10 ppb) |  |  |  |  | 0.42  (0.29–0.61) | < 0.01 | 0.42  (0.29–0.61) | < 0.01 |  |
| CO (per 1 ppm) | 0.46  (0.21–1.00) | 0.05 | 0.45  (0.20–0.98) | 0.04 |  |  |  |  |  |
| O_3_ (per 10ppb) | 0.24  (0.16–0.36) | < 0.01 | 0.24  (0.16–0.36) | < 0.01 | 0.17  (0.11–0.27) | < 0.01 | 0.17  (0.11–0.26) | < 0.01 |  |
| **Akaike information criterion** | 1,632 | | 1,632 | | 1,615 | | 1,615 | | |
| **Coefficient of determination** | 0.33 | | 0.33 | | 0.34 | | 0.34 | | |

A *p*-value < 0.05 is considered statistically significant. Adjusted OR, adjusted odds ratio. CCI, Charlson comorbidity index. CI, confidence interval. NHI, national health insurance. NSAIDs, nonsteroidal anti-inflammatory drugs. NTD, New Taiwan dollar. PM, particulate matter.

***** Models without adjustment for PM10 exposure.

**#** Models without adjustment for PM2.5 exposure.

**Supplementary Table 6. Correlation table for age at first reimbursed biologic initiation and disease duration.**

| **(*n* = 2,920)** | **VIF** | **Age at first NHI-reimbursed biologic initiation** | **Disease duration** |
| --- | --- | --- | --- |
| **Age at first NHI-reimbursed biologic initiation** | 1 | 1 | 0.165 |
| **Disease duration** | 1 | < 0.01 | 1 |

The VIF indicates the level of multicollinearity with the other category, and VIF ≥ 10 is considered significant multicollinearity that needs to be corrected. For the pair of categories, Pearson’s correlation coefficient is presented from the right upper part, and the *p*-value from the left lower part of the table. A *p*-value < 0.05 is considered statistically significant. NHI, national health insurance. VIF, variance inflation factor.
